# The optimal method for improving postural balance in healthy young and older people: specific training for postural tasks encountered in personal physical practice

**DOI:** 10.3389/fphys.2023.1188496

**Published:** 2023-06-28

**Authors:** Thierry Paillard

**Affiliations:** Laboratoire Mouvement, Equilibre, Performance et Santé (UPRES EA 4445), Département STAPS, Université de Pau et des Pays de l’Adour/E2S, Pau, France

**Keywords:** postural balance, postural control, posture, exercise, physical activity, training, specific adaptation, intervention

## Abstract

It is well known that regular exercise or physical activity (training) improves postural balance in healthy young and older subjects, but the optimal exercise or physical activity (i.e., likely to induce the greatest postural improvements) and the context in which it is carried out remain to be explored and determined for each population. The most beneficial adaptations would depend, in particular, on gestural conditions (body position, movement and gesture practiced) and material conditions (nature of the ground surface, sports equipment used, type of environment - stable or changing). In fact, the global postural adaptations induced by training do not result from the transfer between different trained and untrained postural tasks, but are the sum of the adaptations related to each trained postural task in healthy young and older subjects. Based on current knowledge, optimal training programs should include the full range of postural tasks encountered in personal physical practice for each population. To date, the method of implementing progressive postural balance tasks with different degrees of difficulty and instability has been used as the effective method to improve postural balance, but it should not be considered as the reference method. Instead, it should be considered as a complementary method to the one based on specific postural tasks. An intervention strategy is proposed for young and older adults consisting of three different steps (general, oriented and specific/ecologic training). However, some parameters still need to be explored and possibly reconsidered in future studies to improve postural balance in an optimal way.

## Introduction

The most common postures in physical activities and/or sports are the upright posture and the sitting posture. The upright posture is mainly maintained with one or two supports - usually pedal, i.e., standing posture or brachial, i.e., handstand posture—(for example, walking, running, team sports, gymnastics) while the sitting posture is applied with the chest more or less erect (for example, motor sports, cycling, rowing, canoeing). The upright posture being the most variable, is successfully achieved on supports that can be differently configured (separate, grouped, parallel, aligned or staggered, etc.) and oriented (straight, externally or internally rotated, etc.) while the other body segments can also be differently arranged (flexed, extended, aligned, tilted, etc.). Depending on the different positions of support(s) on the ground and placements of body segments in space, postural balance is more or less difficult to maintain. Evidence suggests that the difficulty of postural balance depends on whether the support(s) is (are) brachial or pedal, unique or double, and whether the postural position is dynamic (displacement or deformation of the support base) or static (no displacement or deformation of the support base).

With training and motor experience, individuals can successfully maintain postural balance in increasingly difficult and demanding tasks (Asseman et al., 2004; Asseman et al., 2008). In fact, postural performance (ability to minimize the displacement of the center of gravity or the center of foot pressure as much as possible in quiet stances or ability to maintain body balance in disturbing/destabilizing postural conditions) and postural strategy (characterizes the geometric organization of different body segments and/or neurobiological involvement of the organism for regulating postural balance) are enhanced as a result of the plasticity and adaptability of the postural function and its different sensory, central and motor components generated by training and motor experience (Paillard, 2017). The control of different postures and particular movements regularly repeated during physical activities and/or sports involving specific positions of the supports and body segments generates adaptations of the different components of the postural function (Paillard, 2019). However, exclusive repetitions of specific postures and movements are suspected to induce specific postural adaptations (Giboin et al., 2015). For a given posture and movement, regularly repeated, the postural function generates a singular postural adaptation (Casabona et al., 2016). This means that postural adaptations induced by specific and chronic training (i.e., specific exercises/tasks) involving particular positions of supports and placements of body segments result in no (or very little) transfer to untrained/non-experienced postural and motor tasks (Giboin et al., 2018; Ringhof et al., 2019). However, the influence of the specific gestural and environmental contexts in which postures and movements are carried out and regularly repeated has not yet been clearly identified. To date, detailed analysis of specific postural adaptations to motor and/or sports training and experience (all physical and/or sports activities) has not yet been fully investigated.

This paper aims therefore to analyze the influence of gestural and environmental contexts of regularly repeated postural and motor tasks on postural adaptations. These are examined from a strictly mechanical (i.e., kinematic) point of view and are thus based on the type of activity and/or motor skills practiced, the different positions of the supports on the ground and the placements of the body segments undertaken in space, and the various environmental and material conditions involved. In this way, the data collected would make it possible to identify possible postural specificities that would eventually allow the development of an optimal intervention strategy. Such a strategy would be likely to help sport trainers to optimize motor performance and prevent injuries in athletes, and also help therapists to improve the effects of their programs as well as the measures they implement to prevent falls in frail or older subjects within the framework of specific and intensive sport practices and/or specific postural balance training programs, respectively. Hence, this intervention strategy would fit to all training goals.

## Adaptations of various components of postural function to training and motor experience

As mentioned above, with training and motor experience, the different components of the postural balance function are particularly adaptable (Paillard, 2017).

With regard to sensory components, for most types of physical practice, the contribution of visual information regresses in favor of vestibular, cutaneous and proprioceptive sensory inputs (e.g., Jakobsen et al., 2011; Pau et al., 2012; Kiers et al., 2013; Steinberg et al., 2015). Only certain sports such as, for example, dancing and pistol shooting require significant contribution of visual information to postural regulation in the case of experts, which results from long practice in a very stable environment involving fixed visual cues (Perrin et al., 2002; Herpin et al., 2010; Muelas Perez et al., 2014). In general, it is the contribution of proprioception that increases notably with training and experience for most sports (e.g., Fong et al., 2013; Han et al., 2015). Some activities significantly stimulate the vestibular system through strong linear and angular accelerations and decelerations of the head which develop the capacity to reweight vestibular information and reduce the influence of incongruent vestibular signals with other sensory information, thus allowing for better central integration of vestibular inputs in certain subjects such as gymnasts and fighter pilots (Bringoux et al., 2000; Yang et al., 2015). Other activities practiced barefoot particularly stimulate the plantar cutaneous sole and their regular practice refines the plantar cutaneous sensitivity in relation to activities practiced without shoes (Schlee et al., 2007; Korchi et al., 2019). Moreover, highly experienced individuals are able to prioritize the sensory information detected and change the sensory frame of reference to regulate postural balance under difficult or disturbed postural conditions (Croix et al., 2010; Paillard, 2019). Training can improve the ability to shift the frame of reference, as observed in gymnasts when they adopt the handstand posture (Croix et al., 2010) and may refine overall knowledge of the orientation of the body axis and verticality in physically trained subjects (Paillard et al., 2007a).

Regarding the central component of the postural balance function, with training and experience, the spinal, subcortical and cortical functions are also subjected to adaptations. At the spinal level, training and experience positively modulate the spinal reflexes (e.g., stretch and tendineous reflexes) and the H-reflex of the postural muscles in challenging dynamic postural tasks (Kawaishi and Domen, 2016). At the subcortical level, the automatic compensatory and anticipatory postural adjustments generating the vestibulospinal and reticulospinal reflexes, respectively, are refined and the contribution of cerebellar structures involved in postural regulation are augmented with training and experience (Surgent et al., 2019; Shiozaki et al., 2023; Wang et al., 2023). At the cortical level, a positive linear correlation between the gray matter expansion in the supplementary motor area, the premotor cortex, the frontal and parietal brain areas and postural balance performance has been observed in trained healthy and pathological subjects, both young and old (Taubert et al., 2011; Van Impe et al., 2012; Burciu et al., 2013; Papegaaj et al., 2014). Overall, with training and experience, in the case of moderately difficult postural tasks, the spinal and cortical contributions decrease in favor of the subcortical contribution, while in the case of very difficult/challenging postural tasks, the cortical contribution increases in order to cope with emergencies and to facilitate postural responses to avoid imbalance and/or falling (Payne and Ting, 2020; Saadat et al., 2021). Regarding highly experienced subjects, for a given postural task, the subcortical contribution is further accentuated compared to less experienced subjects (Taube et al., 2007; Taube et al., 2008; Taube et al., 2015).

With regard to the motor component of the postural function, the motor output (muscle strength and power, rate of force development, force variability, and movement control), the joint contribution (mobility, amplitude, stabilization, stiffness), the synergistic muscle command (agonist/antagonist co-activation reduction), and the inter-segmental coordination (phase, anti-phase) are optimized with training and experience (Field, 2011; Nagai et al., 2012; Freyler et al., 2014; Verhoeven and Newell, 2016; Paillard, 2021).

However, taken together, these postural adaptations are relatively global and systemic and cannot be defined as the result of particular training and/or experience (although some functional adaptations of the sensory component are likely to be identified as a result of certain types of physical practice, as indicated above). Thus, it is preferable to analyze particular exercises and motor skills including their execution conditions (gestural and environmental) and to study their postural effects under general and specific assessment conditions in order to identify them accurately.

## Analysis of postural adaptations with respect to gestural, material and environmental factors

### Gestural considerations

#### The influence of the body position in which motor skills are developed

Evidence suggests that different sports and physical activities involve the implementation of an infinite number of motor skills which logically induce different postural adaptations (Zemková and Kováčiková, 2023). Less intuitively, for a given sport, the range of motion (i.e., technical movements) can vary from one individual to another. Subjects develop specific motor skills depending on the type of movements they continuously execute during training. Based on this, it has been suggested that the development of specific motor skills leads to specific postural skills (Paillard, 2014). Postural performance was compared between elite and non-elite gymnasts in different postures more or less specific to the gymnastic activity, i.e., in specifically trained postures and in non-specifically trained postures, focusing on three postural tasks: the upright bipedal being the least specific, the monopedal being more specific and the handstand being very specific- (Asseman et al., 2004). These authors reported that the more trained gymnasts (elites) displayed better postural performance than the less trained gymnasts (non-elites) only in the specifically trained postural task, i.e., in the handstand posture. On the basis of these results alone, we can assume that the level of postural difficulty influences the difference in postural performance in favor of the more trained and better gymnasts, without this necessarily being related to whether the postural tasks were trained or not. This explains why Casabona et al. (2016) developed a protocol based on the foot position that included common and challenging stances, with the level of difficulty changing across the configurations. In these conditions, only one foot configuration was familiar to the dancers. They observed comparable results in dancers and non-dancers during moderate (common stances) and strong (tandem position) increases in difficulty, demonstrating that the appearance of a transfer did not depend on the level of difficulty. In fact, the postural balance of dancers is only favored if the configuration of their feet corresponds to the needs of their practice (duck stance, i.e., feet in extra rotation with heels together and an opening angle of 140° which corresponds to a specific foot configuration in dance). Other authors have since confirmed that expert (elite and professional) dancers present better postural performance than intermediate (non-professional) dancers in dance-like tasks, but not in static everyday tasks (Munzert et al., 2019). In fact, long-term exposure to training gradually links the context to a specific sensory-motor coordination, reducing the chance to develop a transfer of abilities. Overall, postural adaptations are thought to be related to the movement-based learning model which limits the generalization of motor memory to maximize the match between movement control and the demand imposed by a specific context.

#### The influence of technical gestures practiced within a given physical and/or sport activity

For a given physical and/or sport activity, technical gestures may differ from one individual to another and it is thought that they influence induced postural adaptations differently. Paillard et al. (2007b) compared judokas who practiced their favored throwing techniques in a bipedal stance and in a monopedal stance. Their results showed that with two-leg support, the judokas who practiced throwing techniques in bipedal stance were more stable in a standardized dynamic condition than the judokas who practiced throwing techniques in monopedal stance. On the other hand, with one-leg support, the judokas who practiced in monopedal stance were more stable than the judokas who practiced in bipedal stance. Furthermore, within a sports team (e.g., soccer, volleyball and basketball), different players have different roles (i.e., offensive and defensive) and are likely to develop different motor skills and thus, perhaps, different postural abilities (Bizid and Paillard, 2006; Agostini et al., 2013; Pau et al., 2014; Jadczak et al., 2019; Altaweel et al., 2022; Charni et al., 2022). Indeed, postural adaptations differ between offensive, midfield and defensive football players depending on the evaluation conditions considered (Bizid and Paillard, 2006; Pau et al., 2014). In stable postural conditions, offensive soccer players showed better postural performance than defensive soccer players (Pau et al., 2014) while in unstable and spontaneously destabilizing postural conditions, defensive players displayed better postural performance than offensive players (Bizid and Paillard, 2006). This phenomenon could stem from a difference concerning the intention to act/move between defensive and offensive players. Defensive soccer players must react to the motor actions of opposing offensive players and are trained to respond to the challenges of their attacking moves, which develops their compensatory postural adjustments (Bizid and Paillard, 2006). Conversely, offensive players are free in their motor actions by continuously anticipating and creating, but are not trained to respond to actions that are imposed on them, which further develops their anticipatory postural adjustments. All these results suggest that specific technical gestures repeated regularly and frequently during sports practice generate specific postural skills linked to the acquisition of specific motor skills.

#### The influence of the physical and/or sports activities practiced

Knowing that each type of training leads to specific postural adaptations (Zemková and Kováčiková, 2023), Barbado et al. (2016) analyzed postural reactions to mechanical disturbance with subjects who were trained and untrained in terms of postural perturbations. They reported that judokas showed better trunk control after sudden perturbations than kayakers and recreational athletes, as they are frequently challenged by intense sudden disturbances (judokas are subjected to pushes and pulls from their opponents), while kayakers displayed better trunk control in unstable sitting than judokas and recreational athletes, since they are systematically trained in a sitting position on unstable surfaces. Trunk control was not better in judokas and kayakers than in recreational athletes when they were evaluated with tests reflecting no specific skill. Barbado et al. (2016) postulated that postural adaptations can only be detected by specific assessment tests with relevant tasks and tools. Moreover, independently, in postural balance adaptations, each physical and/sport activity may generate particular postures in upright stances, as for instance, asymmetry in body weight distribution (baropodometric variables) on the two feet stemming from asymmetrical orthodox stance in competitive boxers and discrepancy in mean position of the center of feet pressure between bikers (a more posterior position of the center of feet pressure) and runners (Pizzigalli et al., 2013; De Blasiis et al., 2022).

### Material and environmental considerations

#### The influence of the nature of ground surface

Depending on the ground surface, postural balance conditions differ and are likely to induce specific postural adaptations. Surf, being a sport practised in particularly unstable conditions, Paillard et al. (2011) evaluated surfers’ postural performance displaying different sports levels on stable and unstable ground surfaces. Their results showed that the higher the sport level, the better the postural performance of the surfers, only in unstable conditions related to the training postural condition corresponding to the wave movement. However, only postural performance was evaluated, but not postural strategy (defined above), although it would be a rich source of information relating to postural adaptations induced by training. In addition, the effects of specific training based on the nature of the ground surface, i.e., stable or unstable, can only be assessed with subjects trained in either condition. To this end, Powell and Williams (2015) rightly compared the effects of training programs on postural strategy with trained athletes on a stable surface and trained athletes on an unstable surface. They reported that trained athletes on a stable surface exhibited an ascending disto-proximal strategy while trained athletes on an unstable surface displayed a descending proximo-distal strategy. Powell and Williams (2015) specified that athletes trained on a stable surface implemented a reactive feedback-based control strategy whereas athletes trained on an unstable surface implemented a proactive feedforward-based control strategy to anticipate the changes in the unstable condition. In terms of postural strategy, the visual contribution can also differ according to whether the ground surface is firm or soft in combat sports athletes (Jlid et al., 2016). Regular training on a soft ground surface can indeed reduce the proprioceptive contribution and thus increase the visual contribution for postural balance regulation.

#### The influence of sport equipment used

For a given technical gesture practiced, the sport equipment used is likely to influence postural adaptations induced by training. Lion et al. (2009) analyzed postural balance in different cyclists according to the type of bicycle they use and the nature of the environment in which they ride. They observed that road cyclists and mountain bikers developed different postural strategies. Indeed, Lion et al. (2009) found greater advantage was taken of visual information in road cyclists than in mountain bikers when postural condition was primarily dependent on vision, while mountain bikers made more use of proprioceptive information in postural regulation when postural condition was primarily dependent on proprioception. These specific adaptations can be explained by the fact that road cyclists ride on stable and even ground, thus facilitating relevant visual information, whereas mountain bikers ride on unstable and uneven ground which stimulates and enhances proprioception through induced shocks and jolts (Lion et al., 2009). Hence, as mentioned above with pedal supports in the upright posture, the nature of the ground surface during cycling influences the contribution of visual or proprioceptive information in postural regulation after regular training on stable and even ground surfaces or unstable and uneven ground surfaces respectively.

#### The influence of the environment

The nature of the environment in which individuals practice is also likely to influence postural adaptations. Hugel et al. (1999) reported that regular training of dancers in very stable and immobile environments/spaces, including ramps and mirrors, makes them visually dependent. Another activity, such as judo, involves unexpected and permanent postural changes caused by the opponent’s attempts to knock the opponent off balance and onto the ground, thus specifically stimulating and improving proprioception (Perrin et al., 2002).

## Analysis of the transferability of postural adaptations

In order to improve postural balance in athletes and patients, sports coaches and therapists generally elaborate training programs based on progressively difficult postural tasks. However, it is questionable whether the success of increasingly difficult postural tasks really corresponds to an improvement in postural skills in general or whether they correspond to specific postural adaptations to particular postural tasks. Most of the time, postural balance training programs are integrated into overall sports or therapeutic programs in order to improve motor performance (athletes), rehabilitate motor function (patients) or reduce the risk of falling (frail or older subjects). These training programs consist of increasing the difficulty of the postural conditions (support bases, positions, etc.) and modifying the material and environment in order to challenge postural balance.

In young and healthy subjects, slackline training is increasingly used to perform demanding postural tasks. Overall, even if some authors reported that slackline training is likely to generate postural improvement in a standard bipedal stance (Santos et al., 2014), the vast majority of studies dealing with this topic showed that slackline training (over 6 weeks) induced significant task-specific performance improvements but did not affect general postural balance (e.g., Donath et al., 2013; Ringhof et al., 2019). Long-term slackline training—e.g., 12 weeks, two sessions lasting around 40–45 min per week including progressively difficult exercises - corroborated the previous results observed over shorter durations (Giboin et al., 2018; Giboin et al., 2019). Other training methods were tested with various devices, such as, for instance, challenge discs and movable platforms that can be tilted or which generate their own oscillations in all directions (e.g., Giboin et al., 2015; Naumann et al., 2015). Postural adaptations induced by training were limited to test conditions—static or dynamic base of support; monopedal or bipedal stance—and were similar to those that had been undertaken during training (Naumann et al., 2015). These postural adaptations would, moreover, be specific to the balance device used during training, including segmental movement reactions prepared for specific balance disturbances (Naumann et al., 2015). Giboin et al. (2015) suggested that the postural adaptations would be highly task-specific since they observed no transfer even for very similar postural tasks. Overall, balance training induces no effect (or very little) in untrained postural tasks (Giboin et al., 2015; Naumann et al., 2015; Kümmel et al., 2016).

In older subjects, some authors observed that slackline training is likely to improve balance in decontextualized postural tasks on a balance platform (Thomas and Kalicinski, 2016), while other authors found only limited effects (Donath et al., 2016) or even no effects (Glänzel et al., 2022). In fact, a meta-analysis carried out by Glänzel et al. (2022) revealed that the improvement in postural balance is not transferable to other tasks that are similar to the stance induced by practice slacklining (i.e., single leg stance, with the feet side by side, as close as possible). In any event, as in the case of young subjects, the improvements in postural balance resulting from training on slackline generated little or no transfer to other postural tasks in healthy older subjects (when they were able to practice slacklining). This little or no transfer effect in older subjects, would explain why balance training is the mode of physical activity or exercise that has the strongest positive impact on the risk of falling (Wiedenmann et al., 2023).

Overall, the global postural adaptations induced by balance training do not result from the transfer between different postural tasks but result, instead, from the sum of the adaptations linked to each specific postural task practiced/trained in both young and older subjects (Giboin et al., 2015; Naumann et al., 2015; Kümmel et al., 2016; Paillard, 2017; Giboin et al., 2018; Giboin et al., 2019). Beneficial postural adaptations are induced on the basis of previously performed and regularly trained postural tasks, whereas untrained postural tasks have little or no lasting positive postural effect (Serrien et al., 2017).

## Intervention strategy to optimize postural adaptations

In general, in order to improve postural balance, balance training currently includes proactive and reactive exercises in static and dynamic conditions on stable and unstable supports, in the sagittal and frontal planes, with eyes open and closed (Paillard, 2017). The exercises proposed are progressive and based on degrees of difficulty and/or increased instability. However, Blasco et al. (2019) showed that increased difficulty in exercise did not translate into greater improvements. In fact, balance exercises with a higher degree of instability do not necessarily generate greater improvements in postural balance (Blasco et al., 2019). It is not the degree of instability/difficulty but, rather, the degree of specificity that especially influences postural improvements, i.e., the more specific the postural conditions related to balance exercises are in relation to the assessment conditions related to balance tests, the greater the postural improvements. Another fundamental factor in terms of postural adaptations is the amount of physical practice (balance training and/or sport training). Indeed, the number of hours of accumulated practice is a determining factor, since the most highly-trained athletes have better postural balance than other athletes (Paillard, 2017; Paillard, 2019). There is even a dose-response relationship between the amount of balance training (in clinical or sports-related contexts) and the postural improvements in young and older subjects (Lesinski et al., 2015a; Lesinski et al., 2015b).

**FIGURE 1:**
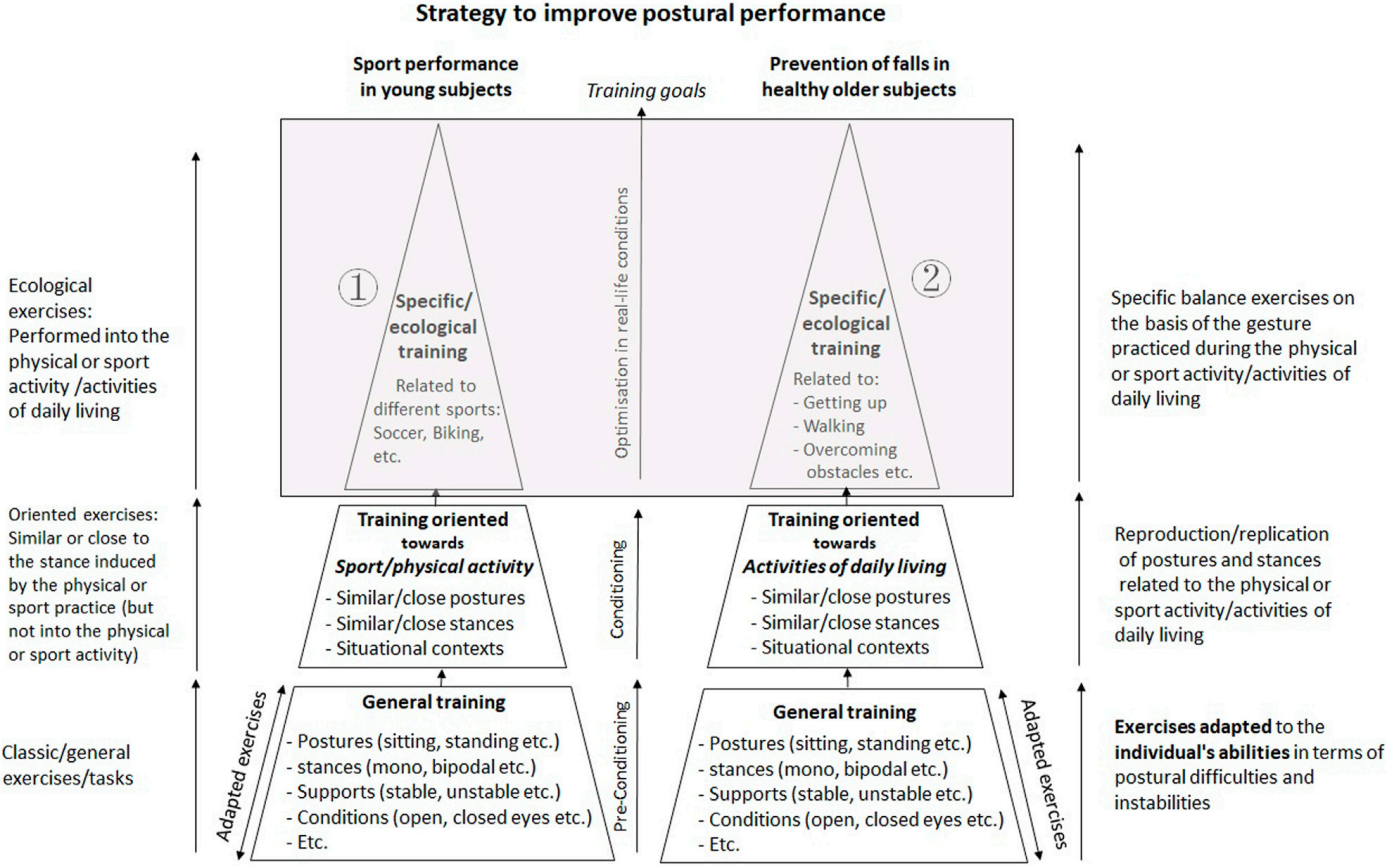
Intervention strategy proposed to optimize postural adaptations in young and older healthy subjects. These are not frail subjects and are capable of performing any exercise independently (without outside help). Obviously, any exercise must be adapted to the individual’s abilities in terms of postural difficulties and instabilities. The content of the gray rectangle in the figure above illustrates the principle of the method based on specific postural tasks and corresponds to the ecological/specific training that follows the oriented training (the body positions and segmental organizations are increasingly close to the specific gestures linked to postural tasks encountered in the personal physical practice for each population) and the general training (different postures, stances, supports and sensorial conditions are undertaken with increasing levels of difficulty). ① Specific/ecological training to improve motor and/or postural performance in young subjects: The balance exercises are based on the implementation conditions of physical and/or sport activities. As examples, soccer players can perform repeated technical movements (e.g., passing, shooting, controlling the ball with different body segments—foot, thigh, trunk, head) in an uninterrupted manner while maintaining postural balance, in a narrow space, on soft ground, on a trampoline, in a monopodal stance, etc. Downhill mountain bike riders can perform balance exercises with a bicycle on wide or narrow supports, stable and unstable supports, in sitting and standing stances, riding on the rear wheel (wheeling), making braking sequences balanced on the front wheel (with the rear wheel off the ground), acrobatic exercises (making aerial turn jumps with the bicycle) etc. ②Specific/ecological training to reduce the risk of falls in healthy older subjects: The balance exercises are based on the implementation conditions of activities of daily living. As examples, older subjects can get up from a chair with different feet positions (feet apart, feet together, semitandem, tandem) on firm and foam supports, with high and low chairs, with or without arm assistance, soft or firm seats, horizontal or inclined seats, with or without a chair back support, etc. Older subjects can also walk barefoot and with large shoes, on a narrow support, on soft or smooth ground, with full tandem foot position, with heel or toe contact, with the head tilted forwards or backwards, with closed eyes, in the darkness, with arms akimbo or crossed, carrying objects, performing a cognitive task (dual-task), with temporal constraints (responses to sound and visual signals), in different acoustic environments (loud noise, pleasant music) etc. They can also overcome obstacles that are wide or narrow, high or low, single or multiple, tight or spread out, etc. As part of a training program aimed at improving postural balance in young and healthy older subjects who have never specifically practiced balance exercises, the intervention strategy should respect the following chronological order: firstly, general training, secondly, oriented training, and thirdly the ecological/specific training. In subjects experienced in balance training, all three training programs (general, oriented and specific/ecological) should be undertaken regularly and alternately to maintain and/or improve postural performance.

In practice, in order to improve postural balance as much as possible, healthy young and older subjects should perform balance exercises that are as specific as possible with respect to the postural conditions involved in professional, military, sport, or artistic environments with a performance objective (i.e., motor performance) and in the simple/ordinary activities of daily life (stand-up, walking, etc.) with a prevention and health preservation objective (i.e., reduction of the risk of falling). For each objective, adapted techniques and methods can be designed to improve postural balance in a specific context (Figure 1).

Moreover, frail and/or older subjects clearly displaying a weakness of the lower-limb muscles need multimodal training with stimulation of neuromuscular function (aimed at improving muscle strength, muscle power/rate of force development, force variability/coordination) combined with stimulation of the postural function to improve postural balance in order to reduce the risk of falling (cf. Paillard, 2021). In any case, muscle strength and especially muscle power should be developed or maintained through the regular practice of physical/sport activities throughout adult life since the force-velocity (force rapidly) quality would be associated with postural performance in healthy older subjects (Orr et al., 2006) and young subjects (Johnson and Woollacott, 2011).

## Conclusion

At present, it seems to be well-established that the postural adaptations induced by balance training do not arise from the transfer between different trained and untrained postural tasks. The beneficial postural adaptations result instead from the sum of the adaptations related to each specific postural task practiced/trained in young and old subjects. In fact, these postural adaptations take place in a specific way according to gestural, material and environmental conditions linked to the postural tasks practiced. Hence, optimal training programs should include the full range of postural tasks encountered in personal physical practice for each population (ecological training). The method of implementing progressive postural balance tasks with different degrees of difficulty and instability (classic method) should be used as a complementary method to the method based on specific postural tasks, but it should not be considered as the reference method.
